# Fractional Control Gate Protocols for Quantum Engines

**DOI:** 10.3390/e28030297

**Published:** 2026-03-05

**Authors:** Elliot John Fox, Taysa Mendes de Mendonça, Ferdinand Schmidt-Kaler, Irene D’Amico

**Affiliations:** 1 School of Physics, Engineering and Technology, University of York, York YO10 5DD, UK; 2 Instituto de Física de São Carlos, Universidade de São Paulo, Sao Carlos 13566-590, SP, Brazil; tmendonca@ifsc.usp.br; 3 QUANTUM, Institut für Physik, Universität Mainz, D-55128 Mainz, Germany; fsk@uni-mainz.de

**Keywords:** quantum computation, quantum protocols, quantum heat engine, quantum thermodynamics, work

## Abstract

Nth-root gates allow for a paced application of two-qubit operations. We apply them in quantum thermodynamic protocols for operating a quantum heat engine. A set of circuits for two and three qubits is compared by considering maximum work production and related efficiency. Our results show that for all circuits considered and most regions of initial parameter space, quantum coherence of one of the qubits strongly increases the maximum work production and improves the system’s performance as a quantum heat engine. In such circuits, coherence is initially imprinted into one of the qubits, improving the overall maximum extractable work. We focus here on the efficiency of such work extraction, assuming the initialisation of the qubits is a free resource. For the novel protocol that employs fractional control gates, work is generated with 84% up to 100% efficiency. Further, we uncover a strong linear correlation between work production and many-body correlations in the working medium generated by these gates.

## 1. Introduction

Quantum thermodynamics (QTD) is a relatively young field that, over recent years, has seen rapid development, becoming an area in which to discuss and challenge well-established concepts, such as the laws of thermodynamics at the quantum scale [1,2,3,4,5,6,7,8,9,10,11,12]. This fast development, coupled with technological advancements in the construction and manipulation of microscopic systems, has facilitated the development of experimental thermal engines that operate outside of the thermodynamic limit [13,14,15,16,17,18,19,20,21,22,23]. Quantum effects, such as coherence and quantum correlations, become increasingly important when trying to accurately describe and model these systems, showing promise for providing advantages over classical systems in thermodynamic processes [5,20,24,25,26,27].

In quantum thermodynamics, quantum resources can be used to cool or heat systems using information processing. The realisation of quantum heat engines (QHEs) demonstrates an application of quantum mechanics for work-producing thermal engines in novel scenarios, and theoretical proposals are implemented across many different models including but not limited to quantum computational protocols [28], laser or photocell QHEs [29,30], magnetically driven QHEs [31], and quantum mechanical adaptations of the Otto cycle [25,32]. The situation where QHEs outperform their classical counterparts [27,33,34] or when quantum resources, such as coherence, correlation, or collective behaviour characterised by many-body interactions, provide an advantage [15,20,27,34,35,36,37,38] is of particular interest. Hence, considering experimental engines facilitating many-body interactions for these new technologies is of significant importance. Quantum computing-inspired protocols for quantum thermal engines have been proposed, with the experimental realisation of many-body systems implementing quantum logic gates achieved utilising platforms such as trapped ions [16,39], Rydberg atoms [40], nuclear magnetic resonance [41], and superconducting qubits [42]. For example, a genuine quantum advantage can be achieved on a quantum processor with correlations between components of the working medium, boosting the extractable work per cycle and producing an efficiency higher than the Carnot standard limit [20].

**Figure 1:**
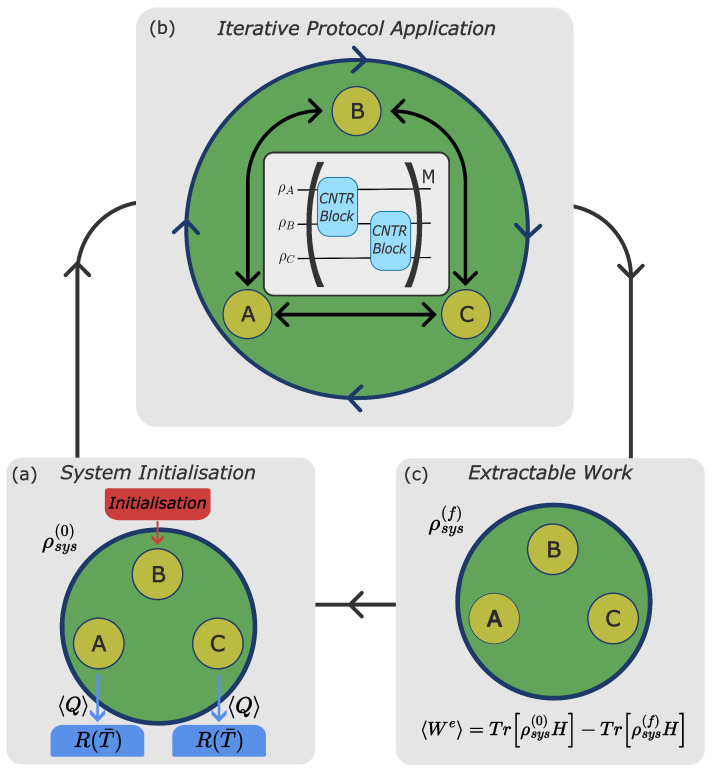
Target operational cycle for the three-qubit quantum heat engine: when qubit-B is initialised to a higher initial energy than qubits A and C, the two-qubit system is recovered with the removal of qubit-C. The operation is cyclic, where after panel (**c**), the system can be reinitialised back to its initial state. To note, in this work, we are interested only in the dynamics of the sequence (**a**–**c**). Three distinct steps are shown: (**a**): system initialisation—Section 2.1; (**b**): iterative protocol application—Section 2.2; (**c**): extractable work. The blue arrows in panel (**a**) between the qubits and reservoirs $R(\bar{T})$ represent heat exchange 〈Q〉 and indicate the flow of energy when initialising qubits A and C as cold qubits; the black arrows in (**b**) show the building of correlations between the qubits mediated by the NRCGs; ‘CNTR Block’ represents different configurations of NRCGs, as shown in Figure 2. Except where otherwise stated, we initialise qubit-B in a pure state with initial coherence. Qubits A and C are initialised in identical Gibbs states using the thermal reservoirs $R(\bar{T})$.

In this work, we introduce an algorithmic QHE composed of a three-qubit working medium whose energy dynamics are driven through the stepped application of quantum computational protocols (Figure 1). Here, extractable work is characterised as a reduction in energy of the total system with respect to its initial state after the protocol is applied. The problem of practically accessing the extractable work in many-body systems for QHEs in proposed theoretical models remains a challenge. Experimentally, we now have the building blocks to realise a QHE, and as the number of qubits available in quantum computers increases, the size of realisable QHEs can increase in turn. The challenge of accessing the extractable work is ongoing, being crucial to the development of a working quantum heat engine. In the present case, the state of our three-qubit system can be accessed through full-state quantum tomography [43,44], which opens the door for the probing and testing of our proposed engine.

**Figure 2:**
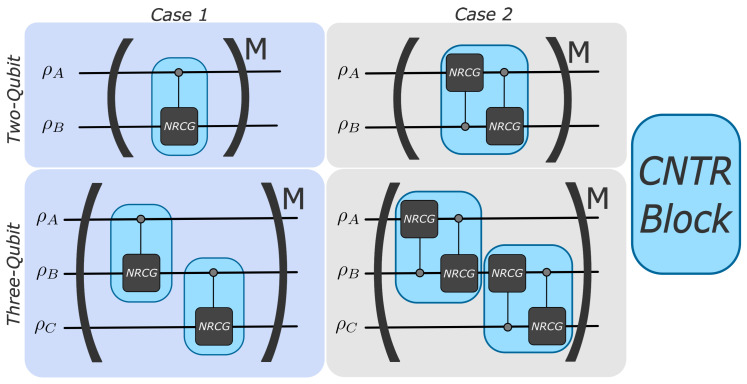
The protocols considered, i.e., Case 1 and Case 2, differ by the set of gates within each ‘CNTR block’ (light-blue boxes), as indicated.

We investigate Nth-root controlled-not logic gates (NRCGs), which, when applied iteratively, act as a trotterisation of controlled-not logic gates, for the implementation of a QHE. NRCGs share similarities with collision models used in other works involving the operation of a QHE [18,45]. Further, exchanging full Pauli gate operations with a fractional iterative protocol has allowed for investigating the presence of quantum friction, i.e., the generation of quantum coherence in the instantaneous energy eigenbasis under a non-permuting protocol [46,47]. The experimental realisation of this protocol has proven that quantum friction induces a violation of the work fluctuation dissipation relation, certifying an additional genuine quantum effect [48].

The NRCG’s stepped approach to the dynamics allows access to intermediate work-producing states that, when using full controlled NOT logic gates, are typically unavailable, allowing for greater control over the quantity of work produced by the engine. We propose and investigate protocols based on four different circuits. When initial states have quantum coherences, we find that this leads to an increase in maximum work production and a larger work-producing parameter region, as compared with initially thermalised qubits. Further, we identify specific regions of heat engine operation and show that when the system is operating in its maximum work-producing regime, there are high values of efficiency, demonstrating the suitability of this approach when considering a quantum heat engine. Finally, a strong linear correlation, quantified by the Pearson correlation coefficient, is uncovered between work production and the quantum mutual information between components of the working medium, generated solely through the application of the NRCG protocols.

## 2. Design and Functioning of the NRCG-Driven Quantum Heat Engine

We consider a system composed either of two (A and B) or of three (A, B, and C) qubits that interact with each other through the use of NRCGs (Figure 2). Our system will undergo one operational cycle that consists of distinct steps: initialisation, where qubits are connected to reservoirs and heat is exchanged, and iterative protocol application, where qubits, now disconnected from the reservoirs, are driven by gates and work is extracted.

Operations involving logic gates may generate entanglement between the component qubits. Figure 1 introduces the target operational cycle of our system as a thermal engine; next, we will describe in detail each step shown.

### 2.1. Initialisation

First, we describe the initialisation step. The component qubits of the working medium are coupled to their respective reservoirs, which initialise or re-initialise them to some initial state. Qubits A and C are initialised in identical thermal states at equal temperatures $\bar{T}>0$ by coupling to the thermal reservoirs $R(\bar{T})$. In the case where qubit-B is initialised in a thermal state, *initialisation* in Figure 1a is performed by the reservoir $R(T_{B})$. It is important to note that there is no initial correlation between any two qubits. The single-qubit thermal state is a Gibbs state defined as [49]

(1) $$\rho_{Gibbsj}\;=\;\left(1/Z_{j}\right)e^{-\beta_{j}H_{j}},$$

where $Z_{j}=\sum_{i}e^{-\beta_{j}\epsilon_{ij}}$ is the partition function, $\beta_{j}={\left(k_{B}T_{j}\right)}^{-1}$ is the inverse temperature parameter, $k_{B}$ is the Boltzmann constant, and $\epsilon_{i}$ are the eigenvalues of the single-qubit Hamiltonian. This is given by

(2) $$H_{j}\;=\;\left(\begin{array}{cc} \epsilon_{1} & 0\\ 0 & \epsilon_{2} \end{array}\right).$$

As reference systems, we consider the ones with each qubit prepared in a thermal state: here, there are no initial quantum coherences. We compare these with the systems in which qubit-B is prepared in a pure state; then, initial quantum coherence will transfer through the qubits when a circuit is applied. This allows for probing how initial quantum coherences affect the system’s capabilities as a heat engine. Qubit-B is initialised in a pure state as $\rho_{Pure}=\left|\psi \rangle \langle \psi \right|$, where $|\psi \rangle =\cos\left(\theta /2\right)|0\rangle +e^{i\phi }\sin\left(\theta /2\right)|1\rangle$ with a value of θ which ranges from 0toπ, while ϕ ranges from 0 to 2π. For this investigation, we will consider the full range of θ and ϕ values. Here, $|0\rangle =\left(\begin{array}{c} 1\\ 0 \end{array}\right)$ is the ground state and $|1\rangle =\left(\begin{array}{c} 0\\ 1 \end{array}\right)$ is the excited state. In this paper, energies are given in units of $\epsilon_{2}$, which is then set to 1 in all calculations.

The total initial Hamiltonian is non-interacting and of the form,

(3) $$H_{sys}=\sum_{j}H_{j}$$

with $H_{j}$ given by Equation (2) and j=A,B and j=A,B,C for two and three qubits, respectively. Equation (3) represents the Hamiltonian for the total system at any time, including at the point of measurement, except when NRCGs are applied, inducing interactions between qubits.

To understand the flow of heat through the system, we first define two quantities: the average internal energy and the extractable work. The average internal energy is quantified with the value $U_{j}$ and has the form $U_{j}=\mathrm{Tr}\left[\rho_{j}H_{j}\right]$, where $\rho_{j}$ is the density matrix of either the component qubits or the total system, and $H_{j}$ is the corresponding Hamiltonian, j=A,B,C for the component qubits or j=sys for the total system.

We now characterise the flow of heat through the system by first discussing the heat exchanged between the qubits and the environment when resetting the system. Qubits A and C are initialised at a different initial energy to qubit-B, allowing for the identification of which components of the working medium are ‘hot’ and ‘cold’, which is important when describing the system as a thermal engine. Explicitly:

- $U_{A}^{\left(0\right)}=U_{C}^{\left(0\right)}<U_{B}^{\left(0\right)}$ identifies qubit-B as the ‘hot’ component, that is, the qubit with the highest initial energy;
- $U_{A}^{\left(0\right)}=U_{C}^{\left(0\right)}>U_{B}^{\left(0\right)}$ identifies qubit-B as the ‘cold’ component, that is, the qubit with the lowest initial energy.

Here $U_{j}^{\left(0\right)}$ is the energy of qubit *j after* initialisation, that is, at the beginning of the driving cycle, and j=A,B and j=A,B,C for two and three qubits. The heat exchanged during (re)initialisation of qubit *j* is then $\langle Q_{j}\rangle =U_{j}^{\left(0\right)}-U_{j}^{\left(f\right)}$, with $U_{j}^{\left(f\right)}$ the internal energy of qubit *j* at the end of the driving part of the operational cycle, that is, just *before* (re)initialisation.

The definition of $\langle Q_{j}\rangle$ allows us to identify the direction of the flow of heat: $\langle Q_{j}\rangle$ is positive when energy is transferred from a reservoir to a component of the working medium during the (re)initialisation phase of the operational cycle.

Further, the system has no intrinsic interaction between the qubits outside of those facilitated by NRCGs, allowing for convenient resetting of the system back to initial conditions.

### 2.2. Protocol Design and Application

The second step of the NRCG-driven quantum heat engine is the iterative protocol application shown in Figure 1b. This is the step in which the working fluid (the qubits), as a closed system, is driven by the unitaries represented by the applied gates, and work is produced. We use the convention [5] that a reduction in the total energy of the system during this step corresponds to the positive extractable work $\langle W^{e}\rangle =-\Delta U_{sys}=-\left\{\mathrm{Tr}\left[\rho_{sys}^{\left(f\right)}H_{sys}\right]-\mathrm{Tr}\left[\rho_{sys}^{\left(0\right)}H_{sys}\right]\right\}$, with $\rho_{sys}^{\left(0\right)}$ the state of the working fluid after initialisation and $\rho_{sys}^{\left(f\right)}$ the state of the working fluid at the end of the driving protocol. This definition of extractable work will be used throughout the paper. We now describe the NRCGs, then the protocols.

Qubits’ correlation, which a CNOT gate may induce, may lead to a quantum advantage in the operation of a quantum heat engine [20]. The NRCG is a method of partially applying a CNOT gate; it is a unitary operation given by [50],

(4) $$\sqrt[{N}]{CNOT_{A,B}}\;=\;\left(\begin{array}{cccc} 1 & 0 & 0 & 0\\ 0 & 1 & 0 & 0\\ 0 & 0 & s & p\\ 0 & 0 & p & s \end{array}\right),\;\;\;\;\;\;\sqrt[{N}]{CNOT_{B,A}}\;=\;\left(\begin{array}{cccc} 1 & 0 & 0 & 0\\ 0 & s & 0 & p\\ 0 & 0 & 1 & 0\\ 0 & p & 0 & s \end{array}\right).$$

Here, the matrix representation of the gates is written in the standard basis {|00〉,|01〉, |10〉,|11〉} and $s=\frac{1}{2}+\frac{1}{2}e^{\frac{i\pi }{N}}$, $p=\frac{1}{2}-\frac{1}{2}e^{\frac{i\pi }{N}}$. The first subscript represents the control qubit, and the second subscript, the target qubit. This type of gate may generate entanglement between two qubits, which adds a level of quantumness to the system, with the standard form of the CNOT gate recovered when N=1.

*N* consecutive applications of an NRCG with the same control and target qubits could be seen as a trotterisation of the CNOT gate, aiming at explicitly implementing the gate, in the limit of a large *N*, as an adiabatic dynamic. In this sense, our protocols give explicit access to intermediate states, e.g., allowing for the opportunity to use states with different degrees of entanglement from the end result of the full CNOT gate. Here, N=15 is chosen, as it shows good access to intermediate states [51] (we note that, for N>15, there is no further appreciable improvement to the system’s performance when considering $\langle W^{e}\rangle$).

A schematic of the building blocks of the protocols is shown in Figure 2: we consider systems of two and three qubits for each protocol and examine two ways of applying interactions. The two-qubit system is recovered with the removal of qubit-C and the second ‘controlled block’ (CNTR block) in Figure 1b. Explicitly:

- Case 1, one gate for each ‘CNTR block’, see left column of Figure 2: Working systems are composed of qubits A and B and A, B, and C. For the two-qubit system, qubit-A is the control qubit, while qubit-B is the target qubit. For the three-qubit system: qubit-A is the control qubit with qubit-B being the target qubit, then qubit-B is the control qubit with qubit-C being the target qubit.
- Case 2, two gates for each ‘CNTR block’, see the right column of Figure 2: Working systems are composed of qubits A and B and A, B, and C. All qubits are either a control or a target qubit over the course of one iteration.

The system is evolved, according to the quantum circuits in Figure 2, with the evolution $\rho_{sys}^{\left(f\right)}=U\rho_{sys}^{\left(0\right)}U^{†}$, where $\rho_{sys}^{\left(0\right)}$ is the initial density matrix of the total system and $\rho_{sys}^{\left(f\right)}$ indicates the total density matrix at the end of the protocol. The unitary U represents the controlled gates specified in Figure 2, as appropriate to each protocol, with NRCGs of the form in Equation (4). For example, $U={\left(\sqrt[{N}]{CNOT_{A,B}}\right)}^{M}$ for Case 1 two-qubit system, with *M* the number of iterations according to the corresponding panel of Figure 2. The simulation of the protocol is implemented by directly applying U in $\rho_{sys}^{\left(f\right)}=U\rho_{sys}^{\left(0\right)}U^{†}$.

We note that only for Case 1 and the two-qubit system, multiples of *N* iterations of the circuit will be equivalent to the application of multiple standard CNOT gates; however, fractions of *N* iterations will allow access to intermediate states in the evolution leading to a CNOT.

These protocols are iteratively applied to the system and can be halted at a chosen number of iterations, which can be optimised for either a maximum or a specific value of $\langle W^{e}\rangle$. Afterwards, the system will then proceed back to the initialisation step.

When qubit-B is initially the most energetic, we can define qubits A and C as the cold components, and qubit-B as the hot component of the working medium. Identifying a heat engine regime can then be achieved with the fulfilment the following conditions

(5) $$\begin{array}{c} \Delta U_{A}\geq 0,\;\Delta U_{C}\geq 0,\;\Delta U_{B}<0,\;\Delta U_{sys}<0, \end{array}$$

with $\Delta U_{j}=\mathrm{Tr}\left[\rho_{j}^{\left(f\right)}H_{j}\right]-\mathrm{Tr}\left[\rho_{j}^{\left(0\right)}H_{j}\right]$ and j=A,B,C. Here $\rho_{j}^{\left(0\right)}$ is the initial and $\rho_{j}^{\left(f\right)}={\mathrm{Tr}}_{\left\{i\neq j\right\}}\left[\rho_{sys}^{\left(f\right)}\right]$ the final state of qubit *j*, the latter being the trace over the degrees of freedom of qubits’ other than *j* of the final state of the system. Conditions (5) are specific to qubit-B being the hot component. While the system does not always operate as a heat engine over all protocols and initial conditions, it does so for the regions corresponding to the highest production of extractable work.

## 3. Plan of the Paper and Anticipation of Main Results

The application of Nth-root gate operations between qubits allows access to the system time evolution in a step-wise manner. Consequently, we consider varying the number of iterations and different initialisations for two different gate building blocks, highlighted in Figure 2.

This paper is composed of two main parts. The first part contains an investigation of the system’s properties as a heat engine, in some detail:

- Optimisation of the initialisation parameters $k_{b}\bar{T}$ for qubits A and C, and θ and ϕ for qubit-B, with the aim of maximising production of extractable work;
- Comparison of results for qubit-B initialised in a pure state against qubit-B initialised in a Gibbs state for identification of advantages obtained from adding quantumness to the system;
- Analysis of the operational cycles leading to maximum extractable work and of their efficiency.

For three out of the four cases and system-size combinations, we find that initial coherence of qubit-B provides an advantage in maximum work production. For all combinations, there is a larger work-producing parameter region when compared with a completely classical initial system. Also, we see that large values of extractable work correspond to large values of efficiency, rendering our system a strong candidate as a quantum heat engine.

The second part utilises the Pearson Correlation Coefficient (PCC) to quantify the correlation between the extractable work and different correlation measures when the system is initialised to produce maximum work. The correlation measures investigated are:

- Mutual information;
- Classical correlations;
- Quantum discord.

Our results demonstrate a strong linear correlation between the mutual information and the extractable work, suggesting that correlations play an important role in work production in a quantum heat engine. Of the mutual information components, it is the classical one that correlates better with the extractable work.

## 4. Results

### 4.1. Temperature Dependence of Work Production

**Figure 3:**
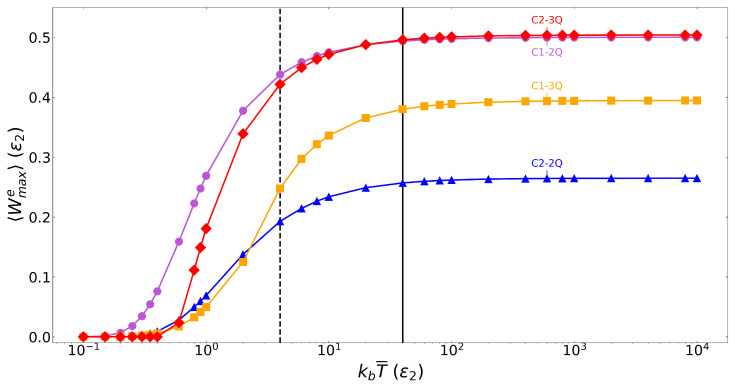
Results demonstrate the $k_{b}\bar{T}$ dependence in units of $\epsilon_{2}$ (x-axis) of the maximum extractable work $\langle W_{max}^{e}\rangle$ in units of $\epsilon_{2}$ (y-axis), where $\langle W_{max}^{e}\rangle$ is recorded over every θ and ϕ combination per value of $k_{b}\bar{T}$. Symbols are the results of the simulations, and corresponding solid lines are to guide the eye. Purple circle: Case 1 two-qubit; blue triangle: Case 2 two-qubit; orange square: Case 1 three-qubit; red diamond: Case 2 three-qubit, as labelled. Black vertical line: $k_{b}\bar{T}=40\epsilon_{2}$. Dashed black vertical line: $k_{b}\bar{T}=4\epsilon_{2}$.

Here, we begin with optimising the system described in Section 2 to identify the temperatures at which to initialise qubits A and C for maximum extractable work $\langle W_{max}^{e}\rangle$. This is defined as the peak value of extractable work across all protocols’ applications and system initialisations. This peak value will occur at some iteration M, where M may differ according to temperature, protocol type, and number of qubits. We note that a small change in the number M of iterations would not vary significantly the work output. Figure 3 shows the temperature dependence of $\langle W_{max}^{e}\rangle$ from all protocols and system sizes, where protocols are applied up to 150 iterations, θ is scanned from 0≤θ≤π, ϕ is scanned from 0≤ϕ≤2π, a comprehensive range of $k_{b}\bar{T}$ is sampled, and $\langle W_{max}^{e}\rangle$ measured for each combination.

Higher initial temperatures lead to larger values of $\langle W_{max}^{e}\rangle$, until saturation occurs for $k_{b}\bar{T}≳40\epsilon_{2}$. This is intuitive; the system is initialised with a higher initial energy, leaving more scope for a larger reduction in energy and a larger amount of work to be produced. Low temperatures show small to no work production until $k_{b}\bar{T}\approx 0.4\epsilon_{2}$ for all but the Case 1 two-qubit system, which produces some amount of work already at $k_{b}\bar{T}\approx 0.1\epsilon_{2}$. Work production then rapidly increases up to $k_{b}\bar{T}\approx 4\epsilon_{2}$, to saturate at higher temperatures to the values of peak work shown in Table 1 for each protocol and system size. This convergence is a result of the thermally initialised qubits approaching maximally mixed states. Because of this, the temperature at which we see the largest $\langle W_{max}^{e}\rangle$ is the same for all cases and system sizes. Figure 3 already demonstrates that the maximum extractable work is not merely connected to the system size and available initial energy, as, depending on the protocol, the two- or three-qubit system may achieve a larger $\langle W_{max}^{e}\rangle$. As this work will focus mainly on maximising work production, in the following, we choose to initialise the system with $k_{b}\bar{T}=40\epsilon_{2}$, which is shown in Figure 3 by the black vertical line. For completeness, an investigation into $k_{b}\bar{T}=4\epsilon_{2}$, signified by the black dashed vertical line, was also performed and showed similar behaviour to that of $k_{b}\bar{T}=40\epsilon_{2}$.

### 4.2. Maximum Extractable Work Dependence on Initial Coherences

**Figure 4:**
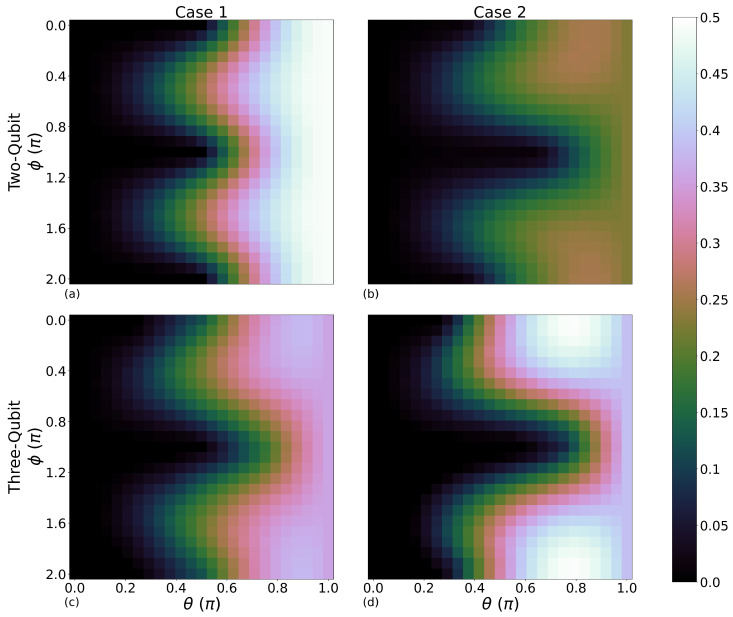
Maximum extractable work $\langle W_{max}^{e}\rangle$ in units of $\epsilon_{2}$ for each {θ,ϕ} combination and 0≤θ≤π (x-axis) and 0≤ϕ≤2π (y-axis); first row: two-qubit systems where columns left to right are cases 1 and 2, respectively. Brighter shades correspond to a greater maximum value of extractable work $\langle W_{max}^{e}\rangle$. Each combination of initial θ and ϕ is run for 150 iterations with $\langle W^{e}\rangle$ recorded at each iteration. Parameters are $\epsilon_{1}=0\epsilon_{2}$ and $k_{b}\bar{T}=40\epsilon_{2}$. Second row: The same parameters as the first row but for the three-qubit systems. (**a**): Case 1 two-qubit, (**b**): Case 2 two-qubit, (**c**): Case 1 three-qubit, (**d**): Case 2 three-qubit.

Following from finding the optimal initial temperature for $k_{b}\bar{T}$ we now investigate the θ and ϕ dependence of $\langle W_{max}^{e}\rangle$. In Figure 4, we are interested in emphasising $\langle W_{max}^{e}\rangle >0$, so that the darkest colour includes all cases for which $\langle W_{max}^{e}\rangle \leq 0$. A range of initialisation values of θ and ϕ are scanned from 0≤θ≤π and 0≤θ≤2π, respectively, with each combination having its respective protocol applied for up to 150 iterations.

Case 1 two-qubit and Case 2 three-qubit systems, Figure 4a and Figure 4d respectively, demonstrate the largest $\langle W_{max}^{e}\rangle$, being the most favourable system combinations for use as an engine. The shape of the work-producing regions provides some insight into the initialisation parameters that lead to large work production for our protocol.

When θ=0.5π qubit-B has the same initial energy as qubits-A and -C: as θ increases, qubit-B becomes the initially hot component of the working medium. Maximum work production always resides in the region where θ>π/2, though work can still be produced when θ<π/2. Table 2 shows the initialisation parameters for peak work production for each case and both two- and three-qubit systems. Apart from the Case 1 two-qubit system, we see that there is also a ϕ dependence on $\langle W_{max}^{e}\rangle$. However, even for the Case 1 two-qubit system, there are values of ϕ for which the work-producing region extends to θ<0.5π. For all cases, the topography of these regions is almost mirrored around ϕ=π, though the largest $\langle W_{max}^{e}\rangle$, by a small margin, is found when ϕ<π. Much like for large values of $k_{b}\bar{T}$ discussed in the previous section, intuitively, larger initial values of θ would produce the most work. Increasing θ increases qubit-B’s initial energy from $\epsilon_{1}=0$ to $\epsilon_{2}$, getting the system close to its maximum possible initial energy. Indeed, large initialisation values of θ are beneficial, but in three out of the four cases the largest values of $\langle W_{max}^{e}\rangle$ are found at θ<π, highlighting the positive role of initial coherences.

### 4.3. Comparison to Initialisation with No Coherences

**Figure A1:**
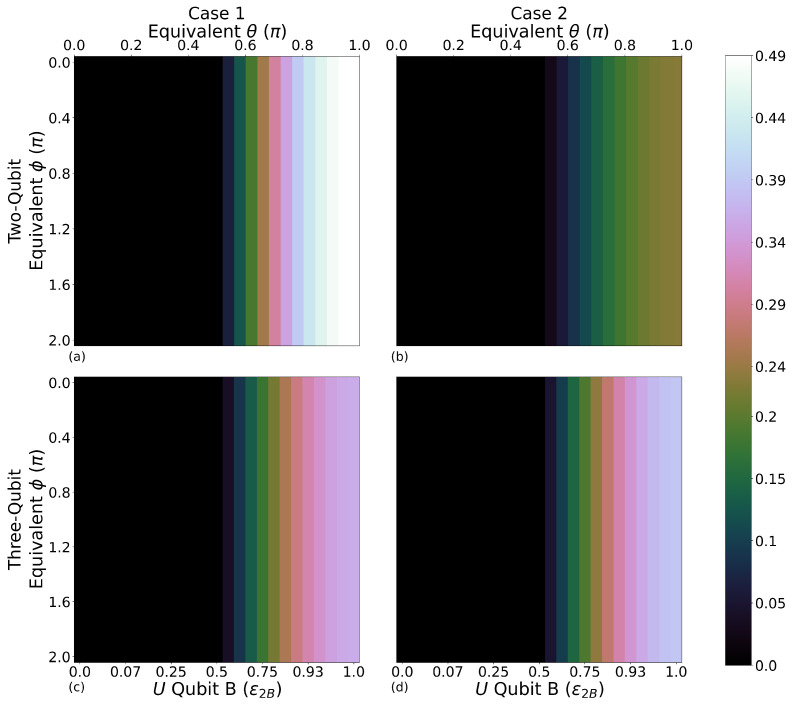
Maximum value of extractable work $\langle W_{max}^{e}\rangle$ in units of $\epsilon_{2}$ for the initial energy of qubit-B $0\leq U^{\left(0\right)}\leq 1$ in units of $\epsilon_{2}$ (x-axis) and corresponding 0≤ϕ≤2π (y-axis); First row: Two-qubit systems where columns left to right are cases 1 and 2, respectively. Brighter shades correspond to a greater maximum value of extractable work. Each combination of initial $U^{\left(0\right)}$ and ϕ is run for 150 iterations. For qubits A and C, $k_{b}\bar{T}=40\epsilon_{2}$. Second row: The same parameters as the first row but for the three-qubit systems. All initial density matrices have no off-diagonal elements, all qubits being initialised in a Gibbs state. (**a**): Case 1 two-qubit, (**b**): Case 2 two-qubit, (**c**): Case 1 three-qubit, (**d**): Case 2 three-qubit.

We now compare the $\langle W_{max}^{e}\rangle$ achieved when qubit-B is initialised with coherence against qubit-B initialised in a thermal state without initial coherence. Effective negative temperatures are included when considering this comparison to achieve equivalent initial ground and excited state populations for qubit-B. The corresponding ‘thermal’ plots to Figure 4 are included in Appendix A, where, in Figure A1, each point in the {θ,ϕ} parameter region has the same initial energy as in Figure 4, and qubit-B is initialised in the Gibbs state corresponding to that energy. The thermal initialisation produces maximum work when qubit-B is initialised entirely in its excited state (θ=π) for all cases and system size combinations. This is in contrast to Figure 4, where for three out of the four case and system size combinations $\langle W_{max}^{e}\rangle$ is achieved when θ<π, and qubit-B has initial coherence.

Figure 4 shows a beneficial ϕ dependence on the work-producing parameter region, as for ϕ≈0.5π and ϕ≈1.5π, the work-producing parameter regions extend to lower values of θ than seen in the completely thermal initialisation. This is true for all cases and system sizes.

The $\langle W_{max}^{e}\rangle$ for the completely thermal case and its increase between the thermal and pure-state initialisation of qubit-B are shown in Table 3, left and right tables respectively. Here, we observe an improvement in $\langle W_{max}^{e}\rangle$ when initialising qubit-B in a pure state for all but the Case 1 two-qubit system, for which the maximum work-producing initialisation parameters with coherences correspond to θ=π. This renders its initial state equivalent to the maximum work-producing initial state for the completely thermal system; hence, there is no increase to the value of $\langle W_{max}^{e}\rangle$. The greatest improvement in maximum work production for the Case 2 three-qubit system, yielding an increase of 25.64%.

We also checked performance against the use of standard—instead of NRCGs—CNOT gates in the protocols: for some parameter regions, NRCGs produce larger peak extractable work than standard CNOT gates, on top of the ability to access intermediate work-producing states not readily accessible by full CNOT gates. Some examples are in Appendix B.

### 4.4. Energy Dynamics When Operating Within a Maximum Work-Producing Regime

**Figure 5:**
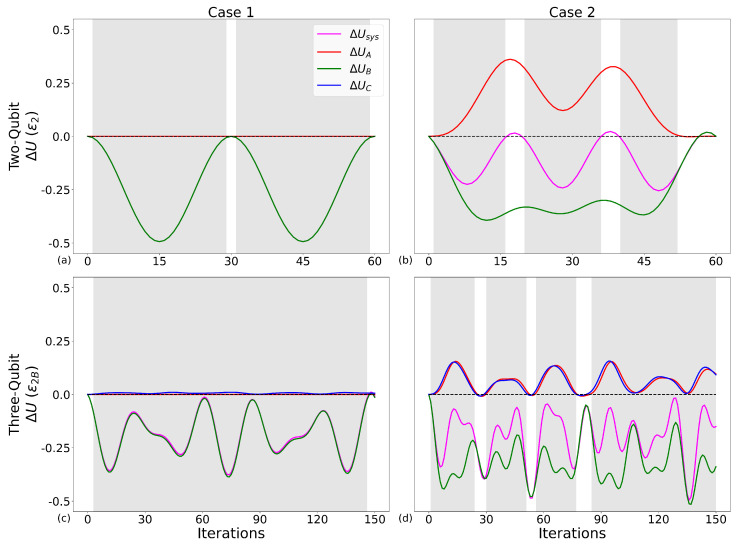
Change in average internal energy ΔU for the total system and component qubits in units of $\epsilon_{2}$ (y-axis) for operational cycles with increasing number of iterations, 0≤ iterations ≤60 for two-qubit systems (first row) and 0≤ iterations ≤150 for three-qubit systems (second row) (x-axis), where columns from left to right are cases 1 and 2, respectively. Corresponding initial θ and ϕ for each panel are shown in Table 2, with ϕ=0.17π for Case 1 two-qubits. Grey shaded regions identify heat engine operation. Same parameters as in Figure 4. red line: $\Delta U_{A}$; green line: $\Delta U_{B}$; blue line: $\Delta U_{C}$; magenta line: $\Delta U_{sys}$. (**a**): Case 1 two-qubit, (**b**): Case 2 two-qubit, (**c**): Case 1 three-qubit, (**d**): Case 2 three-qubit.

In Figure 5, we plot the detailed dynamics of the whole system energy variation $\Delta U_{sys}$, and of its components $\Delta U_{A}$, $\Delta U_{C}$, $\Delta U_{B}$ for initialisations corresponding to the maximum work values shown in Table 2. For Case 1 two-qubits, we choose ϕ=0.17π, for which the work-producing region extends to θ<π/2. The two-qubit systems are run over 60 iterations, and any more, and we observe repeated energy dynamics, while the three-qubit systems are run over 150 iterations. In all panels, qubit-B is initially the most energetic, with its excited state having a higher initial population than qubits A and C. The grey shaded regions in Figure 5 show when the system is operating in a heat engine regime according to the conditions in Equation (5) and will be used for the same purpose in all relevant figures throughout the rest of this work. Case 2, the three-qubit system, shows that producing work, i.e., $\Delta U_{sys}<0$, does not necessarily indicate that the system is operating as a heat engine, with the second largest peak work falling outside the shaded area. However, most of the time, for all cases and system sizes, we generally operate in the heat engine regime. We also see, in general, that an increase in energy of qubits A and C corresponds to a reduction in energy of qubit-B. The exception is the Case 1 two-qubit system. As qubit-A is only ever a control qubit, its initial (local) state is preserved through the application of the protocol, with the system demonstrating no flow of heat from the hot component of the working medium to the cold, though it still produces work. In our protocol we have considered the initialisation of all qubits as a free resource, including the initialisation of qubit-B, which is usually not initialised in a thermal state with positive temperature. In an actual laboratory, initialisation of a *single* qubit in *any* state is not a ‘free’ process in the classical sense. In fact, even the preparation of a positive-temperature single-qubit thermal state requires a (dedicated) protocol very different from connecting it to a reservoir; see, e.g., Box 1 of Figure 1b in [20]. Including qubits’ initialisation energetics in the protocol would affect the computation of work and the related efficiency.

$\Delta U_{A}$ and $\Delta U_{C}$ follow very similar dynamics and, for Case 1, are always almost zero. Their variations for Case 2 are due to the presence of the extra gates. This is reflected in $\Delta U_{sys}$, which, for Case 2, does not always track $\Delta U_{B}$ over the course of repeated iterations. Qubit-B remains the main contributor to the energy dynamics of the total system.

### 4.5. Efficiency

**Figure 6:**
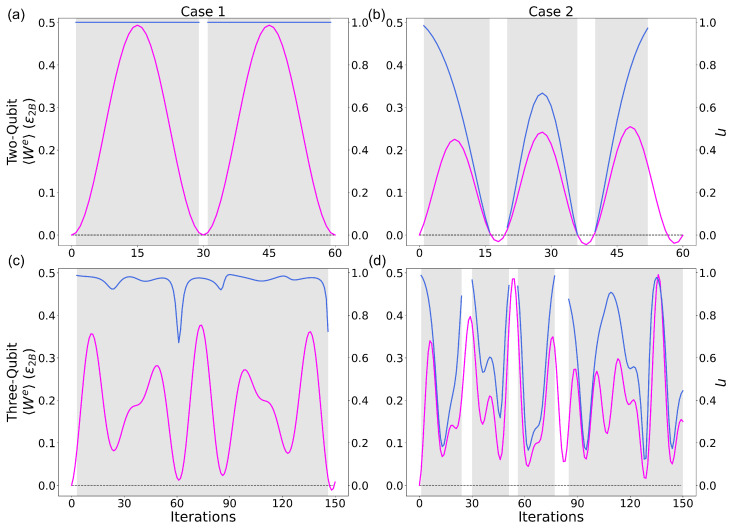
Comparing the extractable work $\langle W^{e}\rangle =-\Delta U_{sys}$ (magenta) in units of $\epsilon_{2}$ (left y-axis) and the efficiency η (blue, right y-axis) for operational cycles with increasing number of iterations, in the case of the two-qubit systems (first row) for 0≤ iterations ≤60 and the three-qubit systems (second row) for 0≤ iterations ≤150, where columns left to right are cases 1 and 2, respectively. Same parameters as in Figure 5. Magenta: $\langle W^{e}\rangle$; blue: η. (**a**): Case 1 two-qubit, (**b**): Case 2 two-qubit, (**c**): Case 1 three-qubit, (**d**): Case 2 three-qubit.

Efficiency is calculated as $\eta =\langle W^{e}\rangle /\langle Q_{H}\rangle$, where $\langle W^{e}\rangle$ is the extractable work and $\langle Q_{H}\rangle =U_{H}^{\left(0\right)}-U_{H}^{\left(f\right)}$ is the energy absorbed by the hot component of the working medium when reinitialised. Comparison of the work and corresponding efficiency is shown in Figure 6, with initialisation parameters corresponding to the maximum work scans in Figure 5. All cases and system sizes are considered. Efficiency is only shown when the system satisfies conditions (Equation (5)) that indicate its operation as a heat engine (grey shaded areas). Table 4 shows the efficiency corresponding to the $\langle W_{max}^{e}\rangle$ in Figure 6 for each case and system size.

Case 1 two-qubit (Figure 6a) differs from all others as $\Delta U_{A}=0$ during the protocol. This results in an efficiency of 1 being achieved, as 100% of $\langle Q_{H}\rangle$ is imparted onto qubit-B and is extractable as work. For all other cases, the efficiency varies as the protocols are applied. Both Case 2 systems show a large variation in the values of efficiency, reflecting the larger energy variation of qubits A and C. Notably, peak values of $\langle W^{e}\rangle$ are marked by peak values of efficiency and/or large efficiency. The more complex efficiency behaviour for Case 2 is to be expected due to the added complexity of the protocol. Case 2 has all qubits, at some point, being the target of an NRCG, whereas Case 1 has one qubit that is never a target and retains its initial energy. This, coupled with doubling the number of gate applications per protocol for the same system size, gives rise to this added complexity.

Comparing with the results in Figure 5, the high efficiency peaks occur when $\Delta U_{A}$ and $\Delta U_{C}$ approach zero and $\Delta U_{B}$ is the dominant component of the work. The Case 1 three-qubit system (Figure 6c) maintains a very high value of efficiency while in the heat engine regime. The efficiency never drops below 0.62 while operating mostly between 0.92 and 0.98, displaying the second most stable efficiency after the Case 1 two-qubit system. Positively, halting the protocol at any of the peak work-producing iterations for this case results in favourable efficiency. Having regions where the engine could be halted that both have large work and efficiency is a positive indicator of the viability of this system’s use as a quantum heat engine.

## 5. Relationship Between System’s Correlations and Extractable Work

We utilise the Pearson Correlation Coefficient (PCC) to quantify the strength of the relationship between a number of correlation measures and extractable work. The PCC is a measure of the linear relationship between two data sets; a value approaching 1 signifies a strong linear correlation, whereas a value approaching −1 signifies a strong anti-linear one. We note that, while the PCC can identify the strength of a linear correlation between two quantities, it does not always indicate the presence of a causal relationship between them. The PCC has the form

(6) $$r_{x,y}=\frac{\sum_{i=1}^{n}\left(x_{i}-\bar{x}\right)\left(y_{i}-\bar{y}\right)}{\sqrt{\sum_{i=1}^{n}{\left(x_{i}-\bar{x}\right)}^{2}}\sqrt{\sum_{i=1}^{n}{\left(y_{i}-\bar{y}\right)}^{2}}},$$

where $x_{i}$ and $y_{i}$ are dataset values, $\bar{x}$ and $\bar{y}$ are the corresponding mean values, and *n* is the length of the data sets *x* and *y*. We are interested in *r* values where r<−0.5 and r>0.5, corresponding to a level of significant correlation.

We now introduce and define the three quantities we will investigate for the remainder of the paper. The mutual information, total classical correlations, and the quantum discord. The mutual information [52] captures all information shared between the components of a chosen bi-partition encapsulating both classical and quantum correlations and is given by

(7) $$I\left(A:B\right)=S\left(\rho_{A}\right)+S\left(\rho_{B}\right)-S\left(\rho_{AB}\right).$$

Here $S\left(\rho_{j}\right)=-Tr\left[\rho_{j}\ln\rho_{j}\right]$ [53,54] is the von Neumann entropy, A,B signifies the components of the chosen bi-partition, and $\rho_{A},\;\rho_{B}$ are the corresponding reduced density matrices traced from the system density matrix $\rho_{AB}$. The quantum discord [55] is a measure of total quantum correlations,

(8) $$D{\left(A:B\right)}_{\left\{\Pi_{j}^{B}\right\}}=\min_{\Pi_{j}^{B}}\left[S\left(\rho_{B}\right)-S\left(\rho_{AB}\right)+S\left(\rho_{A}|\left\{\Pi_{j}^{B}\right\}\right)\right],$$

where $\Pi_{j}^{B}$ are the projective measurements on $\rho_{B}$ and $S\left(\rho_{A}|\left\{\Pi_{j}^{B}\right\}\right)=\sum_{j=u,v}p_{j}S\left(\rho_{A|\Pi_{j}^{B}}\right)$ is the conditional entropy. Here, the post-measurement state of *A* that corresponds to the outcome of the measurement on *B* has the form

(9) $$\begin{array}{c} \rho_{A|\Pi_{j}^{B}}=\left(\Pi_{j}^{B}\right)\rho_{AB}\left(\Pi_{j}^{B}\right)/p_{j},\;\;p_{j}=tr\left[\Pi_{j}^{B}\rho_{AB}\right]. \end{array}$$

The complete orthonormal measurement basis {|u〉,|v〉} is $\left\{|u\rangle =\cos\left(\theta /2\right)|0\rangle +\sin\left(\theta /2\right)e^{i\phi }|1\rangle ,|v\rangle =\sin\left(\theta /2\right)e^{-i\phi }|0\rangle -\cos\left(\theta /2\right)|1\rangle \right\}$ and is used to create projectors for the measurement $\Pi_{u}^{B}=I^{A}⊗|u\rangle \langle v|$ and $\Pi_{v}^{B}=I^{A}⊗|v\rangle \langle v|$, where $I^{A}$ is the identity matrix with dimensionality corresponding to that of subsystem A. To satisfy the minimisation of the discord, measurement is performed for all combinations of 0≤θ≤π and 0≤ϕ≤2π. A full derivation of the discord and its adaptation to the three-qubit case is provided in Appendix C.

Completely classical correlations are also considered. As the discord encapsulates all the quantum correlations in the mutual information, it follows that the remaining correlations are completely classical and given by

(10) $$CC\left(A:B\right)=I\left(A:B\right)-D{\left(A:B\right)}_{\left\{\Pi_{j}^{B}\right\}}.$$

We note that CC(A:B) and $D{\left(A:B\right)}_{\left\{\Pi_{j}^{B}\right\}}$ are not symmetric under the exchange A↔B.

### 5.1. Relationship Between Correlations and Extractable Work

#### 5.1.1. Quantum Mutual Information

**Figure 7:**
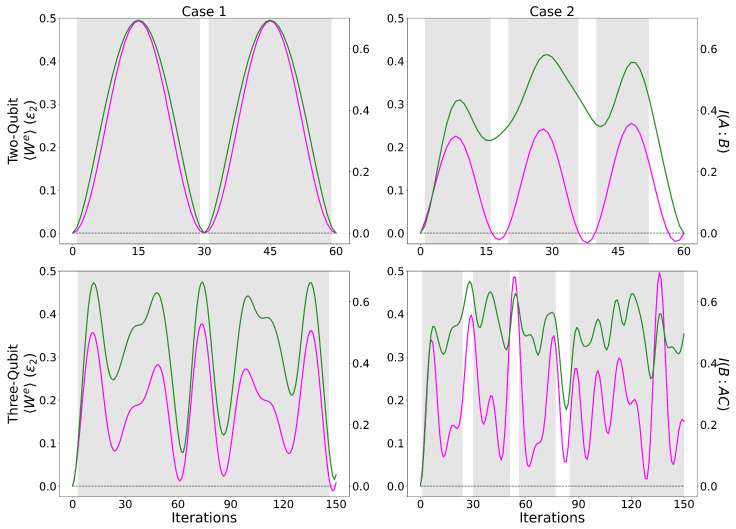
Comparing the extractable work $\langle W^{e}\rangle$ (magenta) in units of $\epsilon_{2}$ (left y-axis) and the mutual information (green) (right y-axis) for operational cycles with increasing number of iterations, in the case of the two-qubit systems (first row) for 0≤ iterations ≤60 and the three-qubit systems (second row) for 0≤ iterations ≤150, where columns left to right are cases 1 and 2, respectively. Same parameters as in Figure 5. Magenta: $\langle W^{e}\rangle$; green: mutual information.

As shown in Figure 4 and described in Section 4.2 and Section 4.3, initial coherence and a finite ϕ were beneficial towards peak work production. We now aim to characterise how correlations between bi-partitions of the working medium, generated through protocols’ applications, are related to the system’s performance. Initial θ and ϕ for Figure 7 are the same as in Figure 5. The selected bi-partition for the two-qubit system is straightforward to choose, with only a single option (*A*:*B*), whereas for the three-qubit system, we have three possible bi-partitions. We choose (*AC*:*B*) due to qubit-B interacting with both qubits A and C directly and to its generally dominant contribution to $\langle W^{e}\rangle$.

PCCs for all cases and system sizes are shown in Table 5. A strong linear correlation between the mutual information and the extractable work is seen in Figure 7 for the Case 1 two- and three-qubit system and the Case 2 two-qubit system. The weakest relationship emerges from the Case 2 three-qubit system, having a PCC of 0.50. However, even with this lower value of PCC, we still find a strong correspondence between the peaks of extractable work and those of the mutual information. For Case 1 (left column), the extractable work is seen to be directly proportional to the mutual information, with larger values of mutual information indicating an equally larger value of work. Case 2 is a more complicated picture. While much like in Case 1, the peaks in mutual information and extractable work are in agreement, the two quantities are strongly correlated but not as directly proportional. This suggests that the additional inverted gates for the Case 2 protocol complicate the work mutual information relationship. Even here, though, data suggest that the building of correlations is beneficial to work production. We find that the mutual information and extractable work for $k_{b}\bar{T}=4\epsilon_{2}$ demonstrate similar linear correlations to the $k_{b}\bar{T}=40\epsilon_{2}$ case, reinforcing the validity of these conclusions.

Strong linear correlation is seen between $\Delta U_{B}$ and the mutual information (Table 5, right) for each protocol and system size. As the mutual information tracks the work, then this is to be expected, with qubit-B generally being the largest contributor to the work for all cases and system, sizes of Figure 5. This is especially true for the Case 1 two-qubit system as the work is only governed by $\Delta U_{B}$. Apart from the sign, there is no difference in PCC for Case 1 when comparing between work and $\Delta U_{B}$; instead, Case 2 for both two- and three-qubit systems shows an increase in PCC of 10% and 18%, respectively, indicating that the strongest correlation is between the mutual information and $\Delta U_{B}$, which itself has the largest impact on the work produced.

As the mutual information is a measure of total correlations between sections of a bipartition, quantifying the individual classical and quantum contributions to the system’s behaviour in terms of work production may provide a more detailed insight. First, we examine the classical correlations, followed by the quantum discord.

#### 5.1.2. Classical Correlations and Quantum Discord

**Figure 8:**
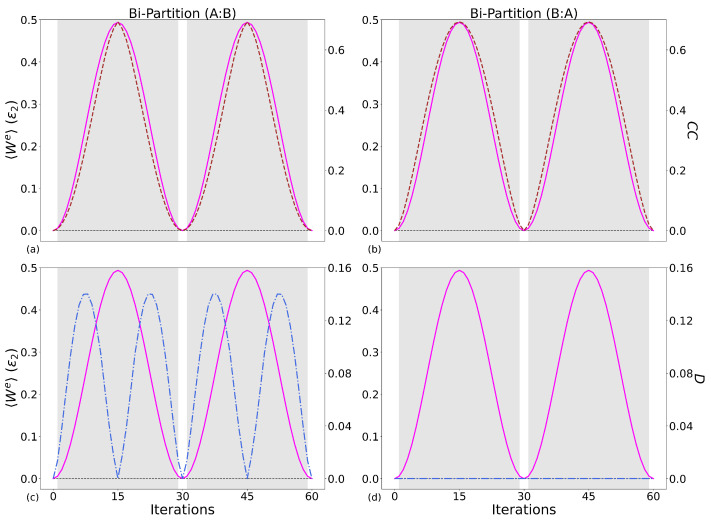
Comparing, for the Case 1 two-qubit system, the extractable work $\langle W^{e}\rangle$ (magenta lines) in units of $\epsilon_{2}$ (left y-axis), the classical correlations (brown dashed lines, top row right y-axis), and the quantum discord (blue dash–dot lines, bottom row right y-axis), for operational cycles with increasing number of iterations, 0≤ iterations ≤60, where columns left to right are the bi-partitions when measuring on $\rho_{B}$ and $\rho_{A}$, respectively. Same parameters as in Figure 5. (**a**): bi-partition (A:B) classical correlations, (**b**): bi-partition (B:A) classical correlations, (**c**): bi-partition (A:B) quantum discord, (**d**): bi-partition (B:A) quantum discord.

**Figure 9:**
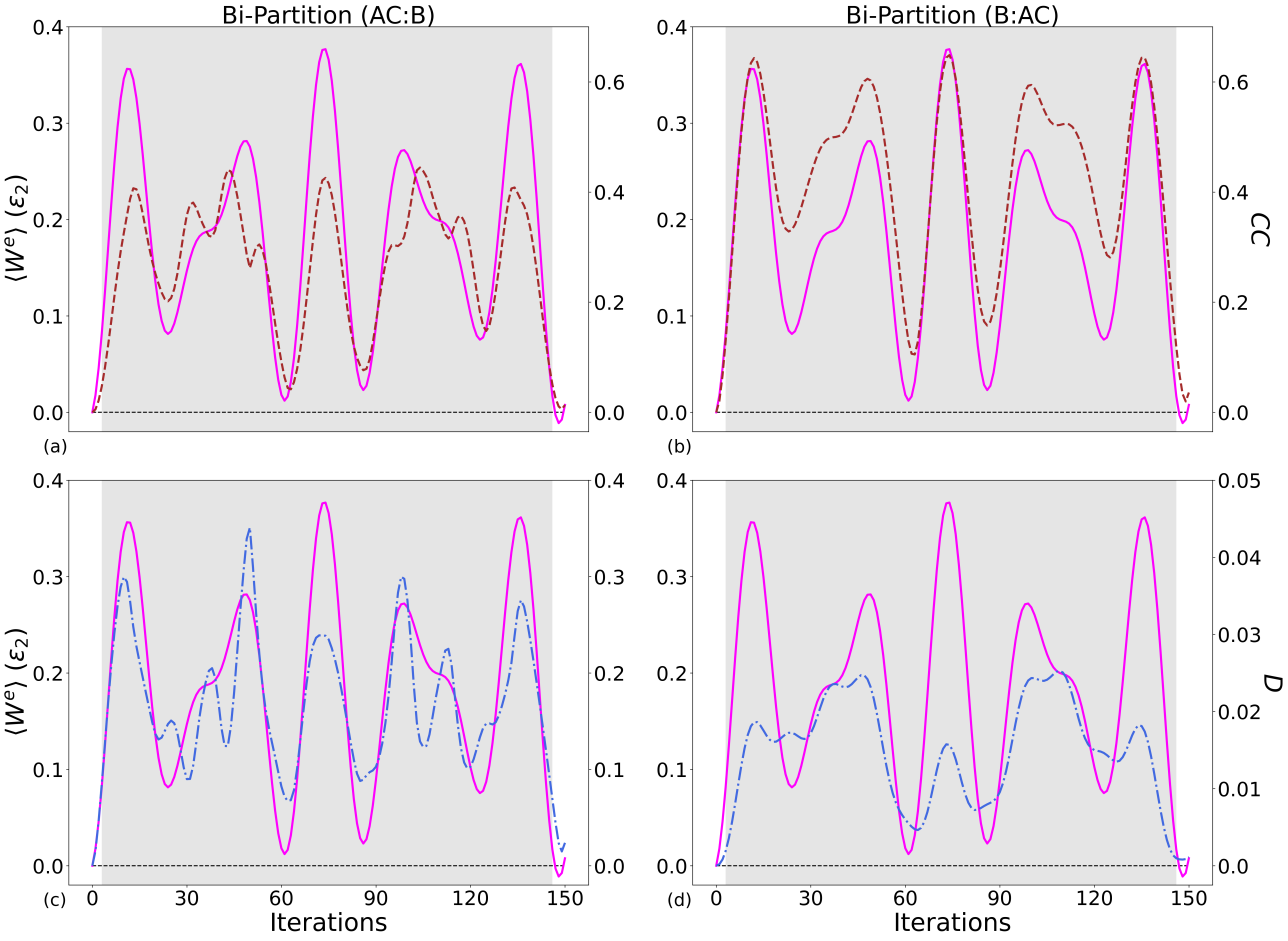
Same parameters as in Figure 8 but for the Case 1 three-qubit system. (**a**): bi-partition (AC:B) classical correlations, (**b**): bi-partition (B:AC) classical correlations, (**c**): bi-partition (AC:B) quantum discord, (**d**): bi-partition (B:AC) quantum discord.

Table 6 and Table 7 contain the PCCs connecting work (left tables) and $\Delta U_{B}$ (right tables) to the classical correlations and quantum discord for all qubit systems and case combinations. Given the bi-partition (*B*:*A*) for two-qubits and (*B*:*AC*) for three, classical correlations correlate to $\langle W^{e}\rangle$ equally or stronger than the mutual information. This can be observed visually by comparing the upper panels of Figure 8 and Figure 9 to the results in Figure 7. This shows that in general, when we generate work, there is a proportional creation of classical correlations between the components of our working medium.

As for the mutual information, Case 2 three-qubit system is showing relatively low PCCs and for both bi-partitions: while some agreement is seen between the peaks of $\langle W^{e}\rangle$ and classical correlations, there is no consistency.

In contrast to the mutual information and classical correlations, no strong linear correlation is seen between the work, $\Delta U_{B}$, and the quantum discord, with the exception of Case 1, the three-qubit system, PCC = 0.86 with bipartition (*AC*:*B*), as shown in the bottom left of Figure 9 and the bottom table of Table 7. Here, *D*(*AC*:*B*) is an order of magnitude greater than *D*(*B*:*AC*), meaning that the information that we can infer about qubit-B from quantum correlations attained by measuring qubits A and C is relatively small. As this is the only system where the quantum discord shows a strong linear correlation with $\langle W^{e}\rangle$, then this is not a universal feature of our system’s performance but system and protocol-specific. For Case 2, the quantum discord has the same order of magnitude as the classical correlations. Related figures are included in Appendix D.

In summary, the mutual information is a strong indicator (but not necessarily the cause) of when our system is producing larger amounts of $\langle W^{e}\rangle$, with work-producing parameter regions generating correlations in the system, with classical correlations playing a leading role. Classical correlations have equal if not better values for the PCC, showing a strong linear correlation with the $\langle W^{e}\rangle$. This is, as expected, mirrored in the PCCs for $\Delta U_{B}$, which is generally the main component driving the production of work. Quantum discord does not show the same strength of linear correlation with $\langle W^{e}\rangle$ as either its classical counterpart or the mutual information, with the exception of the Case 1 three-qubit system, and is zero for bipartition (*B*:*A*). This suggests that, for this type of protocol, the generation and consumption of quantum correlations is, in general, poorly related to work production, being protocol and system-size-specific.

## 6. Discussion

Here, we have shown that initial quantum coherence, solely provided by qubit-B’s initialisation in a pure state, is advantageous to the production of extractable work. Indeed, for three out of four cases and system size combinations, it increases the maximum work production (up to 28.20%) with respect to the initialisation with all thermal qubits. The only case that did not show improvement demonstrated an equal performance. For all combinations, initial quantum coherences also extend the regions of extractable work to θ≤0.5π. These regions show a marked dependence on the azimuthal angle ϕ.

The results presented here in relation to advantages from quantum coherence are corroborated by other works in which coherence can enhance the performance of work extraction protocols [56]. It has also been shown that the amount of work that can be extracted solely from quantum coherence is quantifiable [57], utilising a specific protocol that removes coherence from the system. While this approach can become challenging when considering many-body systems [58], it further confirms coherence as an exploitable resource. Further, while in the present case the internal (quantum) structure of our reservoirs is not considered, it has been shown that coherence present in a heat bath may allow the operation of a single heat bath quantum Photo-Carnot engine [59].

As for any heat engine, classical or quantum, it is desirable to obtain a high efficiency when the engine is producing maximum work. All cases and system sizes demonstrate a high efficiency at peak work, with Case 1 protocols displaying an efficiency always at or close to unity and Case 2 protocols displaying an efficiency tracking more closely the peaks and troughs of work production.

Our results also show the potential advantages of using NRCGs over full CNOT gates in work production. With the trotterised dynamics they induce, NRCG gates provide a more finely tuned and controllable work output for a quantum gate-driven heat engine. In addition, depending on protocol and iterations, NRCGs may lead to higher values of extractable work. Some related results are contained in Appendix B.

The role of quantum correlations in thermal engines is an intricate subject, in part because it is somewhat model-dependent, as discussed by Latune et al. [60]. Typically, related works are focused on advantages gained from initial correlations [20], collective behaviour between *N* identical copies of a heat engine [34], or coherent baths [37]. In this work, we have checked if a relation could be established between the extractable work production and three types of correlation: mutual information, classical correlation, and quantum discord. In our working medium, these are solely generated through the application of our gate-based protocols. To quantify these relationships, we have used the Pearson correlation coefficient. We find a strong linear correlation between the extractable work and both the mutual information and classical correlations. For the latter, the partitions (B:A) and (B:AC) should be considered, with higher linear correlations for cases with lower values of corresponding quantum discord. This, together with other findings, suggests that the production of work is mainly tied to the production of classical correlations for this type of protocol.

We also find, though outside of the scope of the work presented here, that the proposed system can operate in other regimes depending on initialisation parameters, such as a refrigerator and a reverse flow heat engine, which is a uniquely quantum case [61,62]. This naturally leads us towards further investigations and shows potential for the system to be used as a somewhat universal engine, with the operating regime dictated by the needs of the operator. Further investigations could also include the addition of initial correlations between the components of the working medium and correlations between the working medium and the baths in the initialisation step.

## 7. Conclusions

In this paper, we have shown that circuits utilising Nth-root CNOT gates can be advantageously used for quantum heat engines. We evaluate the system’s performance by analysing the maximum work produced when one of the qubits, qubit-B, is initialised in a pure state and find this to be highly advantageous with respect to a system with initial thermal qubits and the same energy. Initial coherences not only improved maximum work production but also increased the size of work-producing parameter regions, extending them to areas corresponding to the initial polar angle of qubit-B smaller than π/2. We also find that the protocol operates at very high to high efficiency when producing its maximum work, further demonstrating its potential use as a heat engine. Finally, we show that correlations described by the quantum mutual information between the components of the working medium demonstrate a strong linear correlation with the extractable work. It is similar for classical correlations when considering a specific partition order, rendering them a key component of work production. The systems and protocols we use are experimentally feasible with current technology.

## Figures and Tables

**Table 1:** Maximum extractable work as converged at large values of $k_{b}\bar{T}$, corresponding to Figure 3.

	〈$W_{max}^{e}$〉 at $k_{b}\bar{T}=10^{4}\epsilon_{2}$	
	Two-Qubit	Three-Qubit
**Case 1**	$0.50\epsilon_{2}$	$0.39\epsilon_{2}$
**Case 2**	$0.27\epsilon_{2}$	$0.50\epsilon_{2}$

**Table 2:** Maximum work values from Figure 4 and their corresponding initialisation parameters for cases 1 and 2. Left: two-qubit; Right: three-qubit.

	Two-Qubit			Three-Qubit		
	θ	ϕ	〈$W_{max}^{e}$〉	θ	ϕ	〈$W_{max}^{e}$〉
**Case 1**	π	any	$0.49\epsilon_{2}$	0.88π	0.24π	$0.38\epsilon_{2}$
**Case 2**	0.83π	0.32π	$0.26\epsilon_{2}$	0.79π	0.08π	$0.50\epsilon_{2}$

**Table 3:** Left: maximum work values for cases 1 and 2 for the two- and three-qubit system with a complete thermal initialisation; right: percentage change in $\langle W_{max}^{e}\rangle$ by introducing initial coherence in qubit-B with a pure state initialisation.

	〈$W_{max}^{e}$〉		Relative Change in 〈$W_{max}^{e}$〉	
	Two-Qubit	Three-Qubit	Two-Qubit	Three-Qubit
**Case 1**	$0.49\epsilon_{2}$	$0.36\epsilon_{2}$	0%	5.56%
**Case 2**	$0.23\epsilon_{2}$	$0.39\epsilon_{2}$	12.04%	28.20%

**Table 4:** Efficiency corresponding to the maximum extractable work, corresponding to Figure 6.

	η at 〈$W_{max}^{e}$〉	
	Two-Qubit	Three-Qubit
**Case 1**	1.00	0.84
**Case 2**	0.97	0.93

**Table 5:** Left: PCC for 〈$W^{e}$〉 versus mutual information corresponding to Figure 7. Right: PCC for $\Delta U_{B}$ versus mutual information.

	〈$W^{e}$〉 Versus Mutual Information			$\Delta U_{B}$ Versus Mutual Information		
	Bi-Partition	Case 1	Case 2	Bi-Partition	Case 1	Case 2
**Two-Qubit**	(A:B)	0.99	0.67	(A:B)	−0.99	−0.77
**Three-Qubit**	(AC:B)	0.92	0.50	(AC:B)	−0.92	−0.59

**Table 6:** Pearson correlation coefficients for the two-qubit systems for the extractable work (left column) and $\Delta U_{B}$ (right column). Top tables: classical correlations; bottom tables: quantum discord.

	〈$W^{e}$〉 Versus Classical Correlations			$\Delta U_{B}$ Versus Classical Correlations		
	Bi-Partition	Case 1	Case 2	Bi-Partition	Case 1	Case 2
**Two-Qubit**	(A:B)	0.99	0.67	(A:B)	−0.99	−0.64
**Two-Qubit**	(B:A)	0.99	0.73	(B:A)	−0.99	−0.38
	**〈$W^{e}$〉 Versus Quantum Discord**			**$\Delta U_{B}$ Versus Quantum Discord**		
	**Bi-Partition**	**Case 1**	**Case 2**	**Bi-Partition**	**Case 1**	**Case 2**
**Two-Qubit**	(A:B)	0.05	0.03	(A:B)	−0.05	−0.41
**Two-Qubit**	(B:A)	0.18	−0.01	(B:A)	−0.18	−0.81

**Table 7:** Pearson correlation coefficients for the three-qubit systems for the extractable work (left column) and $\Delta U_{B}$ (right column). Top tables: classical correlations; bottom tables: quantum discord.

	〈$W^{e}$〉 Versus Classical Correlations			$\Delta U_{B}$ Versus Classical Correlations		
	Bi-Partition	Case 1	Case 2	Bi-Partition	Case 1	Case 2
**Three-Qubit**	(AC:B)	0.81	0.25	(AC:B)	−0.81	−0.47
**Three-Qubit**	(B:AC)	0.92	0.56	(B:AC)	−0.93	−0.57
	**〈$W^{e}$〉 Versus Quantum Discord**			**$\Delta U_{B}$ Versus Quantum Discord**		
	**Bi-Partition**	**Case 1**	**Case 2**	**Bi-Partition**	**Case 1**	**Case 2**
**Three-Qubit**	(AC:B)	0.86	0.42	(AC:B)	−0.47	−0.23
**Three-Qubit**	(B:AC)	0.57	0.16	(B:AC)	−0.57	−0.29

**Figure A2:**
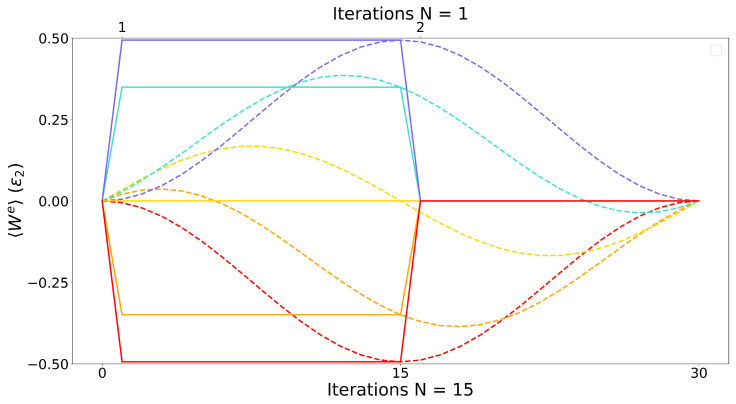
Comparing the extractable work $\langle W^{e}\rangle$ in units of $\epsilon_{2}$ (y-axis) of *N* = 15 (NRCG) and *N* = 1 (full CNOT gate) for the case 1 two-qubit system for two cycles. Same parameters as in Figure 4. ϕ=0.17π. Red: θ=0; orange: θ=0.25π; yellow: θ=0.50π; cyan: θ=0.75π; blue: θ=π. Dashed lines: N=15; Solid lines: *N* = 1.

**Figure A3:**
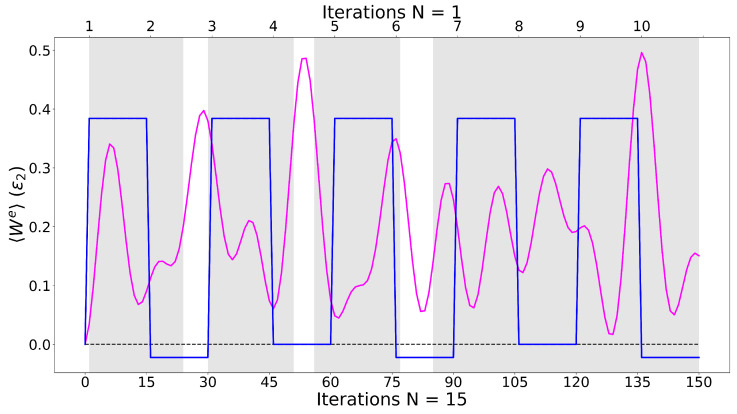
Comparing the extractable work $\langle W^{e}\rangle$ in units of $\epsilon_{2}$ (y-axis) for the NRCG protocol with N=15 (magenta) and with the standard CNOT (N=1, blue) for Case 2, three-qubit systems. Bottom x-axis: 0≤ iterations ≤150 for N=15; Top x-axis: 0≤ iterations ≤10 for N=1. Same parameters as in Figure 5.

**Figure A4:**
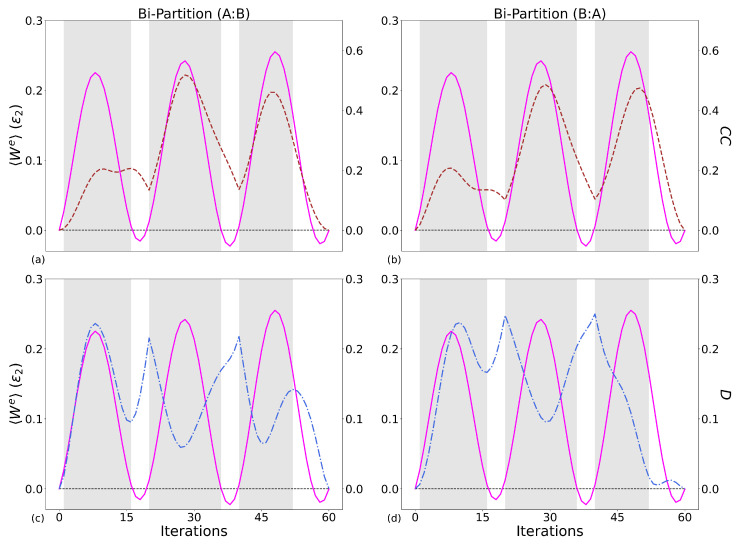
Same parameters as in Figure 8 but for the Case 2 two-qubit system. (**a**): bi-partition (A:B) classical correlations, (**b**): bi-partition (B:A) classical correlations, (**c**): bi-partition (A:B) quantum discord, (**d**): bi-partition (B:A) quantum discord.

**Figure A5:**
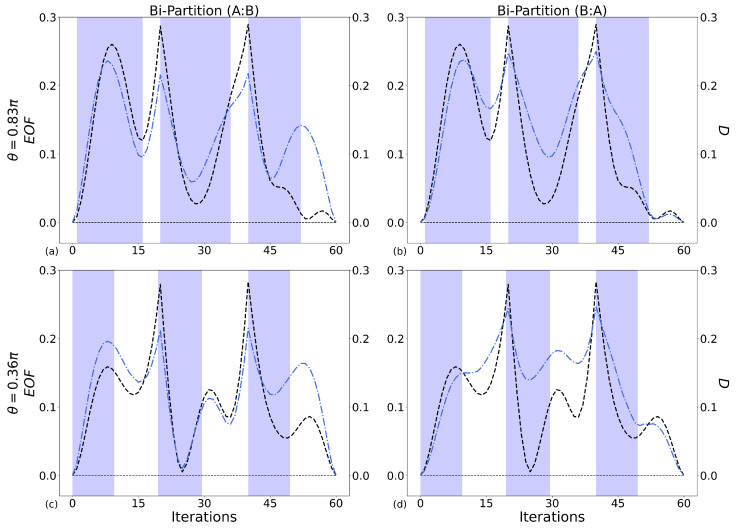
Comparison of the entanglement of formation (left y-axis) and quantum discord (right y-axis) for 0≤ iterations ≤60 (x-axis) for Case 2 two-qubit system. Top row: θ=0.83π; Bottom row: θ=0.36π. Blue shaded regions indicate work production, i.e., regions with $\langle W^{e}\rangle >0$. These include (but do not coincide with) the regions in which the system behaves as a heat engine. Black dashed: entanglement of formation; blue dash dot: quantum discord. (**a**): bi-partition (A:B) θ=0.83π, (**b**): bi-partition (B:A) θ=0.83π, (**c**): bi-partition (A:B) θ=0.36π, (**d**): bi-partition (B:A) θ=0.36π.

**Figure A6:**
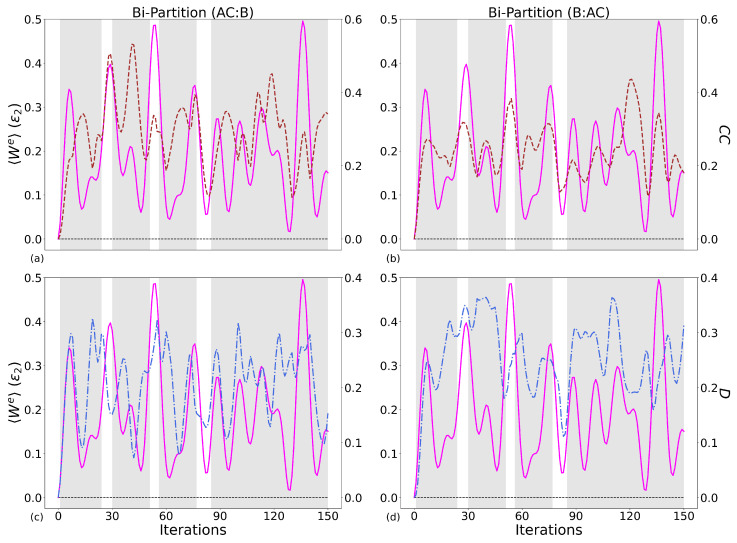
Same parameters as in Figure 8 for the Case 2 three-qubit system. (**a**): bi-partition (AC:B) classical correlations, (**b**): bi-partition (B:AC) classical correlations, (**c**): bi-partition (AC:B) quantum discord, (**d**): bi-partition (B:AC) quantum discord.

## Data Availability

The original contributions presented in the study are included in the article; further inquiries can be directed to the corresponding author.

## Appendix A. Maximum Extractable Work When Qubit-B Is Initialised in a Thermal State

**Table A1:** Maximum values of extractable work when all component qubits are initialised in a thermal state.

	〈$W_{max}^{e}$〉	
	Two-Qubit	Three-Qubit
**Case 1**	$0.49\epsilon_{2}$	$0.36\epsilon_{2}$
**Case 2**	$0.23\epsilon_{2}$	$0.39\epsilon_{2}$

In Figure A1, qubit-B is initialised in a Gibbs state having the same energy *U* (lower x-axis, non-linear scale) as the corresponding pure state characterised by the polar angle θ indicated in the upper x-axis (linear scale). This allows for a direct comparison with Figure 4, where qubit-B is initialised in a pure state. As the off-diagonal elements of a thermal state are zero, in Figure A1, there is no dependence of $\langle W_{max}^{e}\rangle$ on ϕ.

There is an increase in maximum extractable work when the equivalent θ increases from 0.5 to π for all cases and system sizes, demonstrating the requirement for a degree of population inversion for qubit-B. The maximum extractable work is shown in Table A1 and is compared and discussed in the main text.

## Appendix B. Comparison with Circuits Containing Full CNOT Gates

Figure A2 compares $\langle W^{e}\rangle$ between an NRCG and a full standard CNOT gate for the Case 1 two-qubit system with varying θ. Within this appendix, a cycle is defined as *N* iterations of the basic circuit, corresponding to M=N in Figure 1b, to draw a direct comparison between gate types. As only 1 gate is applied in the protocol at each multiple of *N* iterations, both gate types are equivalent. This can be seen as $\langle W^{e}\rangle$ is the same at the end of each cycle, regardless of the initial choice of θ. When 0<θ<π, we start to see where an advantage can be achieved. Within this range, peaks and troughs of $\langle W^{e}\rangle$ for the NRCG are now not aligned with the peaks of the CNOT and are generally advantageous. We see unique behaviour for when θ is initialised at 0.50π (yellow). The full CNOT does not produce any work, which is in contrast to the NRCG, where we do see work production, demonstrating the value of being able to access the intermediate states not readily accessible by a full CNOT. Comparing θ=0.5π to the completely thermal initialisation in Figure A1, where there is also no initial coherence, $\langle W_{max}^{e}\rangle =0\epsilon_{2}$. The thermal and pure-state initialisations of qubit-B are equivalent in initial energy and population of their ground and excited states. The only difference is the initial coherence, with non-zero off-diagonal elements, indicating initial coherence from the non-thermal initialisation coupled with the application of the NRCG, which is what provides this advantage over the full CNOT gates.

When θ=0 or θ=π, any advantages attributed to peak work production disappear. For these values, there is indeed no initial coherence.

Figure A3 shows a comparison of circuits built with NRCGs and N=15 against circuits with full standard CNOT gates (i.e., N=1) when the Case 2 three-qubit system is initialised for maximum work production, as in Figure 5. Here, the grey shaded regions correspond to when the NRCG protocol (magenta line) is operating in the heat engine regime. The protocol is applied for M=10N iterations for both gate types: 150 iterations when N=15; 10 iterations when N=1. Circuits with NRCGs show larger peaks of work production than the ones with full CNOT gates at 30, 55, and 137 iterations, though only the peak at 137 iterations falls within the heat engine regime. The NRCG-based circuit always produces work, while in the same regions, work must be provided to the system for CNOT-based protocols, the corresponding $\langle W^{e}\rangle$ being less than zero. In general, the use of NRCGs demonstrates more control over the quantity of extractable work, exploiting intermediate states not accessible by full CNOT gates. This feature provides a benefit for the quantum heat engine: as the choice of when to halt the application of the protocol resides with the user, an iteration can be chosen that corresponds to the desired value of $\langle W^{e}\rangle$.

## Appendix C. Derivation of the Quantum Discord from the Mutual Information for the One- and Two-Qubit Projective Measurements

Here, we give the full derivation of the quantum discord from the mutual information for both the one and two-qubit projective measurements. The mutual information captures all information shared between the components of a chosen bi-partition, encapsulating both classical and quantum correlations. The mutual information has two equivalent classical expressions,

(A1) I(A:B)=H(A)+H(B)−H(AB),

and

(A2) J(A:B)=H(A)−H(A|B)=H(B)−H(B|A).

The quantum generalisation for the first expression of the mutual information quantifies the total correlations in some bi-partite system which includes both quantum and classical parts [52]. It is symmetric about the bi-partition and has the form,

(A3) $$I\left(\rho_{AB}\right)=S\left(\rho_{A}\right)+S\left(\rho_{B}\right)-S\left(\rho_{AB}\right).$$

Here, the Shannon entropy H(A) [63] has been replaced with the von Neumann entropy [53,54], which is equivalent when using this expression for quantum systems. The von Neumann entropy has the form,

(A4) $$H\left(A\right)=S\left(\rho_{A}\right)=-tr\left[\rho_{A}\ln\rho_{A}\right].$$

The second term in Equation (A2) requires determining the conditional state of subsystem A. The quantum description of J(A:B) is interpreted as the information gained about subsystem A with a measurement on subsystem B and has the form,

(A5) $$J{\left(\rho_{AB}\right)}_{\left\{\Pi_{j}^{B}\right\}}=S\left(\rho_{A}\right)-S\left(\rho_{A}|\left\{\Pi_{j}^{B}\right\}\right).$$

where $S(\rho_{A}|\left\{\Pi_{j}^{B}\right\})$ is the conditional entropy. Maximisation over $J(\rho_{AB})$ encompasses the maximum classical correlations that can be obtained from the chosen bi-partition, with any remaining correlations being completely quantum. Hence, $S(\rho_{A}|\left\{\Pi_{j}^{B}\right\})$ is maximised over the complete orthonormal measurement basis {|u〉,|v〉} and is given by

(A6) $$S\left(\rho_{A}|\left\{\Pi_{j}^{B}\right\}\right)=\sum_{j=u,v}p_{j}S\left(\rho_{A|\Pi_{j}^{B}}\right),$$

which is the sum of the von Neumann entropies of the post-measurement states over the complete set of projective measurements. This yields the conditional entropy of A given the complete measurement $\left\{\Pi_{j}^{B}\right\}$ on B. The complete orthonormal measurement basis {|u〉,|v〉} is,

(A7) $$\{|u\rangle =\cos\left(\theta /2\right)|0\rangle +\sin\left(\theta /2\right)e^{i\phi }|1\rangle ,|v\rangle =\sin\left(\theta /2\right)e^{-i\phi }|0\rangle -\cos\left(\theta /2\right)|1\rangle \}.$$

This basis is used to create projectors for the measurement $\Pi_{u}^{B}=I^{A}⊗|u\rangle \langle u|$ and $\Pi_{v}^{B}=I^{A}⊗|v\rangle \langle v|$ where $I^{A}$ is the identity matrix with dimensionality corresponding to that of subsystem A. The post-measurement, state of A that corresponds to the outcome of the measurement on B has the form,

(A8) $$\begin{array}{c} \rho_{A|\Pi_{j}^{B}}=\left(\Pi_{j}^{B}\right)\rho_{AB}\left(\Pi_{j}^{B}\right)/p_{j},\;\;p_{j}=tr\left[\Pi_{j}^{B}\rho_{AB}\right]. \end{array}$$

With a quantum equivalent now defined for both expressions of mutual information, we can now define the quantum discord. The quantum discord has the form

(A9) $$D{\left(A:B\right)}_{\left\{\Pi_{j}^{B}\right\}}=I\left(A:B\right)-\max_{\Pi^{B}}J{\left(A:B\right)}_{\left\{\Pi_{j}^{B}\right\}},$$

where $D{\left(A:B\right)}_{\left\{\Pi_{j}^{B}\right\}}$ is the quantum discord, I(A:B) is the mutual information, and $\max_{\Pi^{B}}J{\left(A:B\right)}_{\left\{\Pi_{j}^{B}\right\}}$ are the classical correlations. Finally, inserting Equations (A3) and (A5) into Equation (A9), we obtain

(A10) $$D{\left(A:B\right)}_{\left\{\Pi_{j}^{B}\right\}}=\min_{\Pi^{B}}\left[S\left(B\right)-S\left(AB\right)+S\left(A|\left\{\Pi_{j}^{B}\right\}\right)\right].$$

To satisfy the minimisation criteria, the discord is calculated over 0>θ>π and 0>ϕ>2π. Quantum discord for the three-qubit system requires an expansion of our measurement basis to cover two qubits simultaneously due to the bi-partition of one and two qubits. This measurement basis is

(A11) $$\{|u\rangle |u^{\prime }\rangle ,|u\rangle |v^{\prime }\rangle ,|v\rangle |u^{\prime }\rangle ,|v\rangle |v^{\prime }\rangle \},$$

where

(A12) $$\begin{array}{c} |u\rangle =\cos\left(\theta /2\right)|0\rangle +\sin\left(\theta /2\right)e^{i\phi }|1\rangle ,|v\rangle =\sin\left(\theta /2\right)e^{-i\phi }|0\rangle -\cos\left(\theta /2\right)|1\rangle \\ |u^{\prime }\rangle =\cos\left(\theta^{\prime }/2\right)|0\rangle +\sin\left(\theta^{\prime }/2\right)e^{i\phi^{\prime }}|1\rangle ,|v^{\prime }\rangle =\sin\left(\theta^{\prime }/2\right)e^{-i\phi^{\prime }}|0\rangle -\cos\left(\theta^{\prime }/2\right)|1\rangle . \end{array}$$

Projectors are created in the same way as for the single-qubit measurement,

(A13) $$\begin{array}{c} \Pi_{uu^{\prime }}^{AC}=|u\rangle \langle u|⊗I⊗|u^{\prime }\rangle \langle u^{\prime }|,\;\Pi_{vu^{\prime }}^{AC}=|v\rangle \langle v|⊗I⊗|u^{\prime }\rangle \langle u^{\prime }|,\\ \Pi_{uv^{\prime }}^{AC}=|u\rangle \langle u|⊗I⊗|v^{\prime }\rangle \langle v^{\prime }|,\;\Pi_{vv^{\prime }}^{AC}=|v\rangle \langle v|⊗I⊗|v^{\prime }\rangle \langle v^{\prime }|. \end{array}$$

Measurement is performed for all values of 0≤θ≤π, 0≤ϕ≤2π, $0\leq \theta^{\prime }\leq \pi$, and $0\leq \phi \leq 2\pi^{\prime }$.

## Appendix D. Classical Correlations and Quantum Discord for Case 2

The classical correlations and quantum discord for the Case 2 two-qubit system are shown in Figure A4; the quantum discord is non-analytic at 21 and 41 iterations, abruptly reducing in value, showing a cusp. At these iterations, we observe the system re-entering the heat engine regime, which is signified here by $\langle W^{e}\rangle <0$ evolving to $\langle W^{e}\rangle >0$. This is the sole change occurring at these iterations, which removes this protocol and system size from the heat engine regime, suggesting that for this case, as we increase iterations, we cross the work-producing threshold, quantum correlations are consumed, accompanied by a sudden rise in classical correlations.

In Figure A5, we show the entanglement of formation and quantum discord for the Case 2 two-qubit system to emphasise the similarity in behaviour. The entanglement of formation has the form [64]

(A14) $$EOF_{AB}=-xlog_{2}x-\left(1-x\right)log_{2}\left(1-x\right),$$

where $x=\frac{1-\sqrt{1-\tau }}{2}$, $\tau =[max\left(\lambda_{1}-\lambda_{2}-\lambda_{3}-\lambda_{4},0\right)]$, $\lambda_{i}=\sqrt{\epsilon_{i}}$, and $\epsilon_{i}$ are the eigenvalues of the matrix $\rho_{AB}\bar{\rho_{AB}}$. Here $\rho_{AB}$ is the reduced density matrix of sites A and B, and $\bar{\rho_{AB}}$ is the spin-flipped $\rho_{AB}$, so $\bar{\rho_{AB}}=\left(\sigma_{y}^{A}⊗\sigma_{y}^{B}\right)\rho_{AB}^{*}\left(\sigma_{y}^{A}⊗\sigma_{y}^{B}\right)$. The PCC values shown in Table A2 demonstrate a strong linear correlation between the discord and the EOF.

The cusps at 21 and 41 iterations discussed previously are mirrored in the entanglement of formation (see Figure A5), suggesting that it is the sudden reduction in entanglement that drives a reduction in the quantum discord. This is not only seen for the parameters of Figure A4 (first row of Figure A5) but also for other initial θ for qubit-B (e.g., second row of Figure A5), suggesting this to be a consistent behaviour for the protocol. In comparison, for Case 1, two-qubit, or either of the three-qubit systems, no entanglement of formation is produced for any of the possible bi-partitions, and no cusps are seen, rendering this a unique feature.

**Table A2:** PCCs for the quantum discord and the entanglement of formation when the system operates in a maximum work-producing regime and when we operate in the mode of work symmetry.

	Entanglement of Formation Versus Quantum Discord	
	Bi-Partition (A:B)	Bi-Partition (B:A)
θ=0.83π	0.85	0.91
θ=0.36π	0.81	0.78

Finally, we examine the different correlation types for the Case 2 three-qubit system in Figure A6. It can be seen that the evolution with protocol application of the correlations is complex, being challenging to identify a relationship between the $\langle W^{e}\rangle$ and both the classical correlations and the quantum discord. Some peaks of the classical correlations correspond to peaks of the $\langle W^{e}\rangle$; this can be seen in the PCC value for the (B:AC) bi-partition (Table 5), being greater than that of the mutual information (Table 7). Even so, there is no clear observable linear correlation between $\langle W^{e}\rangle$ and the two types of correlation that contribute to the mutual information.
